# The Status, Need, and Influence of Dental Prosthetics on Oral Health-Related Quality of Life in the Geriatric Population: An Epidemiological Survey

**DOI:** 10.7759/cureus.27637

**Published:** 2022-08-03

**Authors:** Jayant Prakash, Pushpraj Singh, Divya Dubey, Mahesh Suganna Golgeri, Shaista Haleem, Ashok Bhati, Shivakumar G C

**Affiliations:** 1 Dentistry, Sadar Hospital, Muzaffarpur, IND; 2 Department of Dentistry, Government Medical College, Shahdol, IND; 3 Department of Prosthodontics, Sri Sai College of Dental Surgery, Vikarabad, IND; 4 Department of Prosthodontics, Riyadh Elm University, Riyadh, SAU; 5 Department of Restorative Dentistry, College of Applied Medical Sciences, Riyadh Elm University, Riyadh, SAU; 6 Department of Preventive Dental Sciences, College of Dentistry, Jazan University, Jazan, SAU; 7 Department of Oral Medicine and Radiology, People’s College of Dental Science and Research Centre, Bhopal, IND

**Keywords:** epidemiology and biostatistics, prosthetic status, prosthetic need, ohrqol, geriatric population

## Abstract

Background

An epidemiological survey was conducted among the geriatric population to determine the status and need for dental prosthetics and their influence on oral health-related quality of life (OHRQoL).

Methodology

The study population consisted of 270 patients aged 60 or older. All pertinent demographic information, clinical data on prosthesis status and need, and the Oral Health Impact Profile (OHIP)-14 questions to assess OHRQoL were collected using a pretested structured questionnaire written in the patient’s native tongue. In addition, a type 3 oral examination was performed on a sample of the geriatric population.

Results

The OHRQoL was found to be unaffected by the prosthetic status. However, there was a strong correlation between the elderly study population’s OHRQoL and the need for a prosthetic. Of each variable in OHRQoL, physical pain, discomfort when eating, and loss of taste were the most affected in this population.

Conclusions

The prosthetic needs of the study population must be given high priority as there are many unmet needs. The concerned health departments need to seriously consider increasing facilities with an affordable system. There is a lack of appreciation for OHRQoL. However, given that a satisfactory clinical assessment of the mouth does not always indicate good oral health status, the justification for evaluating dental care with respect to oral well-being is compelling.

## Introduction

Aging is a ubiquitous, imminent, and indelible biological process which influences every individual in one or another form [1]. In most advanced countries, population growth is a debate and, as a result, the aging population is quite visible. By 2025, there will be approximately 600 million people who are 60 years of age or older, a figure that is predicted to double [2]. By 2050, there will be two billion elderly people, with the majority residing in industrialized countries (80%) [3]. The geriatric population in Jharkhand (a state in India) is less than the national average of 8% which is quite alarming [4].

It is the responsibility of healthcare professionals to work toward not only increasing life expectancy but also, perhaps, more importantly, making the latter years of life more beneficial and enjoyable [5,6]. Because oral health influences appearance, communication, and quality of life, it has significant biological, psychological, and social repercussions. Dental caries, periodontal disease, tooth loss, wasting illnesses, edentulism, xerostomia, oral malignancies, etc. are just a few of the oral health issues facing the elderly in India. Complete tooth loss, also known as being entirely edentulous, is a common and irreversible health issue in elderly people and indicates poor dental health [7]. Dietary habits are impacted by edentulism, especially concerning restricted masticatory function, eventual weight loss, and personal satisfaction from burning calories. The elderly may have the greatest number of obstacles to receiving oral health care due to financial issues, a lack of treatment options, and numerous chronic and psychiatric disorders. For any planning, knowing the status is essential, and the measure of prosthetic status and needs will help understand the reality the elderly are facing today [8,9].

The World Health Organization (WHO) describes health as a source of living rather than a state when health definition is considered. This understanding has resulted in the creation of tools such as the health-related quality of life (HRQoL) metric, which describes how health affects people’s capacity to function and the perception of their level of well-being considering the physiological, intellectual, and sociocultural factors [10]. In dentistry, a major effort has been made to develop instruments for determining the oral aspects of HRQoL considering different aspects of everyday living. The multiple dimensions and subjectivity of standard of living are rooted in each person’s personal basis of comparison. It is now acknowledged as a reliable standard for evaluating patients in all fields of cognitive and emotional healthcare, to consider how people perceive their place in life in relation to the belief and value of life in the context and concerning the objectives, aspirations, principles, and fears [11-13].

## Materials and methods

This cross-sectional survey was conducted to determine the prosthetic needs and oral health-related quality of life (OHRQoL) among the geriatric population of Ranchi, Jharkhand, Eastern India. This study was conducted among adults 60 years of age and beyond. The relevant review board examined and approved the protocol’s methodological and regulatory components, and the requisite clearance was acquired (IRB number IEC/IRB/FDS/2022/02/04). Written informed consent was obtained from every participant prior to data collection. This survey was conducted over six months from February 2019 to June 2019.

A convenience sample of elderly participants aged 60 years and above constituted the study population. The study area was geographically divided into eight zones, and from each zone, a total of 30 subjects were selected. After around 30 individuals from one zone had been questioned and studied, the next zone was recruited. A total of 270 subjects constituted the population under our review. The kappa co-efficient value for reliability for prosthetic status and prosthetic needs was 1 and 0.97, respectively. These values reflect the high degree of conformity in observations.

Using a precision of 5%, a design effect of 1, and an alpha error of 5%, the sample size was calculated to be roughly 250. To account for the additional variables, the sample size was rounded to 270. By way of a personal interview and examination by the certified examiner, a specific questionnaire was exclusively developed for documenting all the necessary and relevant general information and clinical results. With the help of the World Health Organization’s (WHO) Oral Health Assessment form, prosthetic status and needs were recorded [14]. Aseptic precautions were employed prior, during, and after the examination. On every survey day, a total of 25-30 respondents were questioned and examined, and the time it took to collect data on each respondent varied from five to ten minutes.

The data were collected and fed into a spreadsheet. The significance level was set to 5% when this was later converted to SPSS software version 18 (IBM Corp., Armonk, NY, USA). Mean, standard deviation (SD), numbers (n), and percentages (%) were used to express continuous and categorical variables, respectively, followed by an analysis of variance (ANOVA) analysis of the variables.

## Results

The data obtained from the study were subjected to statistical analysis. The results of the various parameters considered in this study are shown in Table 1.

**Table 1:** Variables of the study participants.

Variables analyzed		Gender					
		Male		Female		Total	
		n	%	n	%	n	%
Age	60–69 years	62	37.3	104	62.7	166	61.5
	70–79 years	35	46.1	41	53.9	76	28.1
	80–89 years	8	33.3	16	66.7	24	8.9
	90–99 years	1	25	3	75	4	1.5
Education	No formal education	5	17.9	23	82.1	28	10.4
	Primary education	19	27.9	49	72.1	68	25.2
	Secondary	49	42.2	67	57.8	116	43
	Graduation	23	51.1	22	48.9	45	16.6
	Post-graduation	10	76.9	3	23.1	13	4.8
Occupation	Present	16	80	4	20	20	7.4
	No occupation/Retired	90	36	160	64	250	92.6
Diet	Vegetarian	21	42.9	28	57.1	49	18.1
	Mixed diet	85	38.5	136	61.5	221	81.9
Systemic illness	Present	68	36	121	64	189	70
	Absent	33	51.6	31	48.4	64	23.7
	Not aware	5	29.4	12	70.6	17	6.3
Present consumption of tobacco	Yes	28	87.5	4	12.5	32	11.9
	No	78	32.8	160	67.2	238	88.1

In this study, a total of 270 participants constituted the study population, of whom 39.3% were males and 60.7% were females, with ages ranging between 62 and 94 years. The mean age group was 66.4. A majority (61.5%) belonged to the 60-69-year age group. Overall, 43% of the subjects had completed secondary education, followed by primary education with 25.2%, and most of the subjects were retired (92.6%). Mixed diet was seen among 81.9% of the subjects, and only 18.1% were vegetarians. Major illnesses were present in 70% of the study participants, while 6.3% were not aware of it. Regarding the consumption of tobacco, only 11.9% were current consumers. Table 2 shows the prosthetic status and prosthetic needs for both upper and lower jaws.

**Table 2:** Distribution of the study participants according to the prosthetic status and needs in the upper and lower jaws.

Prosthetic status and needs		Male		Female		Total	
		n	%	n	%	n	%
Prosthetic status: upper jaw	No prosthesis present	89	84	132	80.5	221	81.9
	Any prosthesis present	17	16	32	19.5	49	18.1
Prosthetic status: lower jaw	No prosthesis present	94	88.7	136	82.9	230	85.1
	Any prosthesis present	12	11.3	28	17.1	40	14.9
Prosthetic needs: upper jaw	No prosthesis needed	45	42.5	55	33.5	100	37
	Need for one-unit prosthesis	12	11.3	14	8.5	26	9.6
	Need for multi-unit prosthesis	6	5.7	8	4.9	14	5.2
	Need for a combination of one and/or multi-unit prosthesis	38	35.8	74	45.1	112	41.5
	Need for full prosthesis	4	3.8	10	6.1	14	5.2
	Not recorded	1	0.9	3	1.8	4	1.5
Prosthetic needs: lower jaw	No prosthesis needed	44	41.5	63	38.4	107	39.6
	Need for one-unit prosthesis	14	13.2	13	7.9	27	10
	Need for multi-unit prosthesis	4	3.8	6	3.7	10	3.7
	Need for a combination of one and/or multi-unit prosthesis	40	37.7	67	40.9	107	39.6
	Need for full prosthesis	3	2.8	13	7.9	16	5.9
	Not recorded	1	0.9	2	1.2	3	1.1

An overall 81.9% and 85.1% had no prosthesis present in the upper and lower jaw, respectively, and 18.1% (upper) and 14.9% (lower) had some prosthesis present. Among males, 84% in the upper jaw and 88.7% in the lower jaw had no prosthesis present. Among females, 80.5% in the upper jaw and 82.9% in the lower jaw had no prosthesis present. When the results were compared with prosthesis status between males and females, it was found to be statistically not significant for upper and lower jaws (p = 0.125, chi-square = 7.452). When the results were compared between males and females, it was found to be statistically significant (p < 0.05). Similarly, when prosthetic needs were compared between upper and lower jaws, it was found to be significant (p < 0.05).

The OHIP-14 questionnaire was used to assess the OHRQoL in the study population, and an overall mean of 0.87 and standard deviation of 0.421 was found. The majority of the study population did not show much of an impact on OHRQoL except for aching pain, uncomfortable eating, and loss of taste (Table 3)

**Table 3:** Individuals marked according to OHIP-14 scores. OHIP: Oral Health Impact Profile

OHIP-14 items	Distribution of response (%)			Mean	Standard deviation
	Always	Sometimes	Never		
Have you had events where your teeth, mouth, or dentures caused you to have any issues in speaking or saying words?	3	10	87	0.82	0.396
Have you noticed that issues with your teeth, mouth, or dentures have made your sense of taste even worst?	2	23	75	0.71	0.452
Have you had any terrible oral discomfort?	3	35	62	0.64	0.532
Have issues with your teeth, mouth, or prostheses made it hard for you to eat certain foods?	5	25	70	0.69	0.508
Have issues with your teeth, mouth, or dentures made you feel self-conscious?	2	8	90	0.88	0.358
Have issues with your teeth, mouth, or dentures made you feel strained?	4	6	90	0.90	0.302
Has your diet been unsatisfactory as a result of dental, oral, or denture issues?	4	14	82	0.83	0.396
Have issues with your teeth, mouth, or dentures forced you to skip meals?	5	12	83	0.85	0.372
Have issues with your teeth, mouth, or dentures made it difficult for you to unwind?	3	6	91	0.92	0.272
Have issues with your teeth, mouth, or dentures caused you to feel a little embarrassed?	0.5	2	97.5	0.98	0.252
Have issues with your teeth, mouth, or dentures caused you to become a little irritable around other people?	2	3	95	0.96	0.192
Have issues with your teeth, mouth, or dentures prevented you from performing your regular duties?	0.5	2.5	97	0.97	0.294
Have issues with your teeth, mouth, or dentures rendered you completely unable to operate effectively?	0.8	4.2	95	0.95	0.259
Have you ever felt that issues with your teeth, mouth, or dentures made life in general less pleasurable?	0.6	2.4	97	0.97	0.247

The OHIP scores of both jaws (p = <0.05) are shown in Table 4.

**Table 4:** Multiple comparisons of OHIP-14 and upper prosthetic needs of the study population. OHIP: Oral Health Impact Profile; ANOVA: analysis of variance

OHIP-14 components	Prosthetic needs	Upper arch		Lower arch	
		Mean	Standard deviation	Mean	Standard deviation
Functional limitation	No prosthesis	0.81	0.04	0.88	0.06
	One-unit prosthesis	0.83	0.03	0.80	0.03
	Multi-unit prosthesis	0.87	0.02	0.83	0.02
	A combination of one and multi-unit prosthesis	0.71	0.19	0.73	0.18
	Full prosthesis	0.59	0.03	0.58	0.02
	Total	0.76	0.11	0.76	0.12
Physical pain	No prosthesis	0.73	0.01	0.63	0.09
	One-unit prosthesis	0.64	0.06	0.75	0.00
	Multi-unit prosthesis	0.70	0.09	0.70	0.05
	A combination of one and multi-unit prosthesis	0.48	0.12	0.50	0.06
	Full prosthesis	0.44	0.02	0.42	0.04
	Total	0.60	0.14	0.61	0.13
Psychological discomfort	No prosthesis	0.89	0.02	0.91	0.02
	One-unit prosthesis	0.95	0.02	0.95	0.00
	Multi-unit prosthesis	0.88	0.06	0.86	0.06
	A combination of one and multi-unit prosthesis	0.86	0.02	0.89	0.09
	Full prosthesis	0.86	0.03	0.85	0.04
	Total	0.88	0.04	0.90	0.03
Physical disability	No prosthesis	0.85	0.01	0.85	0.00
	One-unit prosthesis	0.88	0.01	0.88	0.02
	Multi-unit prosthesis	0.77	0.00	0.77	0.05
	A combination of one and multi-unit prosthesis	0.83	0.03	0.83	0.08
	Full prosthesis	0.75	0.01	0.75	0.01
	Total	0.82	0.05	0.82	0.06
Psychological disability	No prosthesis	0.93	0.04	0.94	0.02
	One-unit prosthesis	0.98	0.02	0.98	0.05
	Multi-unit prosthesis	0.99	0.01	0.99	0.03
	A combination of one and multi-unit prosthesis	0.95	0.06	0.95	0.09
	Full prosthesis	0.93	0.02	0.92	0.12
	Total	0.96	0.04	0.96	0.02
Social handicap	No prosthesis	0.93	0.01	0.94	0.01
	One-unit prosthesis	0.98	0.02	0.95	0.00
	Multi-unit prosthesis	0.99	0.00	0.99	0.01
	A combination of one and multi-unit prosthesis	0.98	0.02	0.98	0.07
	Full prosthesis	0.89	0.10	0.89	0.05
	Total	0.96	0.04	0.94	0.06
Handicap	No prosthesis	0.91	0.01	0.93	0.01
	One-unit prosthesis	0.99	0.01	0.97	0.00
	Multi-unit prosthesis	0.97	0.02	0.97	0.00
	A combination of one and multi-unit prosthesis	0.95	0.08	0.96	0.06
	Full prosthesis	0.88	0.05	0.88	0.04
	Total	0.95	0.08	0.93	0.04
		(ANOVA between groups p = 0.001)		(ANOVA between groups p = 0.001)	

## Discussion

This is the first cross-sectional study evaluating the prosthetic status and prosthetic needs and how they may influence the OHRQoL among the elderly population residing in Ranchi, Eastern India.

Oral health of an individual has a known downside with aging, ranging from gingival issues to more complex periodontal problems with or without loss of teeth. However, good care can slow down these damages, thus preventing and prolonging oral health and general health, which, in turn, has a great influence on the quality of life. The common risk factors known for tooth loss have been dominated by dental caries, periodontal diseases, trauma, drugs, consumption of tobacco, and a few other congenital developmental disorders [14]. All of these can be prevented to some extent by taking good care, but, in reality, all individuals face these common issues and end up having prosthodontic-related issues. Understanding the oral health status of the elderly population helps us plan better and deliver healthcare services; however, it is the most neglected sector in a country like India where oral health is never a priority at any level. Hence, this study seeks to understand how dentures can influence the general well-being of an individual.

The population aged 60 years and above was selected for the study. This geriatric population was the target for the study as the WHO defines geriatric patients who are aged 60 years and above. Many textbooks and articles contraindicate and prefer 65 years and above; however, practically, this would make little difference with life expectancy falling in many countries [15,16].

The majority of the subjects (61.5%) belonged to the 60-69-year age group and 28.1% belonged to the 70-79-year age group, which was similar to the study by Albin et al. (2016) [3]. Healthcare education plays a crucial role in understanding preventive or service-oriented clinical care. In general, good education can prevent many diseases as awareness makes a difference. This study population had 43% of secondary education, followed by primary education by 25.2%, which is similar to the study by Albin et al. (2016) [3]. Occupation of the individual dictates the purchasing power of an individual regarding health services or any other needs in life. However, in this population, 92.6% were retired or had no jobs. It is generally understood that the retirement age in India stands at 60 years, and those with jobs may have had private jobs, which is similar to the study by Albin et al. (2016) [3]. Diet has a direct impact on health. A balanced diet and the need for protein are essential to having a healthy body and mind. In this study, 81.9% were on a mixed diet. Moreover, systemic illness of the individuals was also evaluated, and it was found that 70% of the population was aware of the illness and 6.3% were not aware. Old age generally has one or the other health issues which justify the situation [3]. Lastly, regarding the consumption of tobacco, a majority (88.1%) did not consume tobacco in any form. This may reflect the awareness of the ill effects of tobacco.

In this study, 81.9% of people had no prosthesis in their upper jaw, and 85.1% had none in their lower jaw. This percentage was on lower strata than that of Shenoy and Hegde and comparable to institutionalized variables in the study by Deogade et al. [17,18]. This result was low when compared to Shah et al. [19] and Ettinger et al. [20], which included individuals from a varied range of ages. Overall, 16.5% of the individuals (18.1% and 14.9% in the upper and lower jaws, respectively) had one or more prostheses present, which was higher than the estimate provided by Ettinger et al. [20] (15.6%). With a p-value of 0.125, it was determined that there were no statistically significant differences in upper or lower jaw prosthesis status between males and females. These findings conflicted with those reported by Shah et al. and were similar to those of Shenoy and Hegde. The widespread use of prosthetics, regardless of need, may be attributed to a few factors, including lack of knowledge, lack of excitement for aesthetics, financial constraints, and scarcity of dental services.

Contrary to the DI National Oral Health survey conducted in 2004, the demand for upper jaw prostheses was slightly higher (63%) than that for the lower jaw (60.4%) [21]. The total prosthetic requirements, however, were comparable to those reported by Mann [22] and Shetty et al. [15].

Similar results have been reported in other global studies [23,24]. The higher needs of female patients than those of their male counterparts were explained by their reliance, higher levels of illiteracy, and prevalence of unemployment [25].

The prosthetic needs of the upper jaw among males were 57.5% which was slightly low when compared to the lower jaw (58.5%). Similar investigations were done by Montal et al. [26], Shah et al. [27], and Shigali et al. [28], though the results obtained were higher in our research. While among females it was more in the upper jaw (66.5%) than the lower jaw (61.5%). When the results were compared between gender, it was found to be statistically significant with a p-value of <0.05. Similarly, when prosthetic needs were compared between upper and lower jaws, it was found to be significant with a p-value of <0.05. The lower jaw with a thick bone density among males could be attributed to its lesser damage while among female systemic disorders associated with osteoporosis, the menstrual cycle could affect the bone density, and thus jaw support could be less for the tooth to be retained.

Analyzing OHRQoL scores, which represent a person’s degree of well-being, is helpful for determining the potential effects of oral problems [29]. OHRQoL is a self-reported indicator of a patient’s current and recent health. It has been added to mortality and morbidity as a reliable indicator of outcomes and has grown to be crucial when making medical and dental decisions. Oral health has an impact on how people perceive their overall health state, and this is particularly important for elderly people. After accounting for factors such as overall well-being and wealth, it was observed that oral conditions that typically affect older populations, such as missing teeth, dry mouth, and mastication difficulties, are associated with lower quality of life. Socioeconomic level, frequency of dental visits, treatment-seeking behavior, challenges with everyday activities, and the severity of systemic disorders are other factors that can have an impact. There is evidence to suggest that elderly people with poor dental health have less positive social contact and lower self-esteem in general, which, in turn, have a negative impact on their general well-being and state of health. OHRQoL has been shown to positively affect happiness after controlling for socioeconomic and demographic characteristics [30-32]. OHRQoL, which also takes into consideration patients’ assessments of their oral health, targets variables affecting oral health in a patient’s daily routine. In this situation, it is necessary to understand whether expectations, adaptation, and normalization are taken into account, as well as to identify what constitutes and who is affected by an important change in the quality of life. These problems must be tackled in the field of dentistry in addition to being addressed in general medicine. A larger multicentric study is needed to assess the real picture with a wide population range.

## Conclusions

The study population’s dental well-being was unaffected by their prosthetic status. However, the research population’s requirement for upper and lower jaw prosthetics was strongly correlated to oral health, with physical pain, difficulty in eating, and loss of taste sensitivity being the most severely impacted. The explanation for the extensive prosthetic requirements was the absence of dental care. In addition, prosthetic rehabilitation among the geriatric population was expensive and unaffordable for many.
